# Regulation of IFN response gene activity during infliximab treatment in rheumatoid arthritis is associated with clinical response to treatment

**DOI:** 10.1186/ar2912

**Published:** 2010-01-22

**Authors:** Lisa GM van Baarsen, Carla A Wijbrandts, François Rustenburg, Tineke Cantaert, Tineke CTM van der Pouw Kraan, Dominique L Baeten, Ben AC Dijkmans, Paul P Tak, Cornelis L Verweij

**Affiliations:** 1 Department of Pathology, VU University Medical Center, De Boelelaan 1118, 1081 HZ, Amsterdam, The Netherlands; 2 Current address: Department of Clinical Immunology & Rheumatology, Academic Medical Center, University of Amsterdam, Meibergdreef 9, 1105 AZ, Amsterdam, The Netherlands; 3 Department of Clinical Immunology & Rheumatology, Academic Medical Center, University of Amsterdam, Meibergdreef 9, 1105 AZ, Amsterdam, The Netherlands; 4 Department of Molecular Cell Biology & Immunology, VU University Medical Center, Van der Boechorststraat 7, 1081 BT, Amsterdam, The Netherlands; 5 Department of Rheumatology, VU University Medical Center, De Boelelaan 1117, 1081 HV, Amsterdam, The Netherlands

## Abstract

**Introduction:**

Cross-regulation between TNF and type I IFN has been postulated to play an important role in autoimmune diseases. Therefore, we determined the effect of TNF blockade in rheumatoid arthritis (RA) on the type I IFN response gene activity in relation to clinical response.

**Methods:**

Peripheral blood from 33 RA patients was collected in PAXgene tubes before and after the start of infliximab treatment. In a first group of 15 patients the baseline expression of type I IFN-regulated genes was determined using cDNA microarrays and compared to levels one month after treatment. The remaining 18 patients were studied as an independent group for validation using quantitative polymerase chain reaction (qPCR).

**Results:**

Gene expression analysis revealed that anti-TNF antibody treatment induced a significant increase in type I IFN response gene activity in a subset of RA patients, whereas expression levels remained similar or were slightly decreased in others. The findings appear clinically relevant since patients with an increased IFN response gene activity after anti-TNF therapy had a poor clinical outcome. This association was confirmed and extended for an IFN response gene set consisting of *OAS1*, *LGALS3BP*, *Mx2*, *OAS2 *and *SERPING1 *in five EULAR good and five EULAR poor responders, by qPCR.

**Conclusions:**

Regulation of IFN response gene activity upon TNF blockade in RA is not as consistent as previously described, but varies between patients. The differential changes in IFN response gene activity appear relevant to the clinical outcome of TNF blockade in RA.

## Introduction

Cytokines are key regulators of pathogenic processes in a variety of inflammatory and autoimmune diseases. Major roles for both tumor necrosis factor (TNF) and type I interferon (IFN) have previously been demonstrated. Type I IFN (IFNα/β) plays an important role in systemic lupus erythematosus (SLE) [1]. Evidence for the role of IFN in SLE came from the induction of disease during IFNα/β treatment and circulating IFN inducers [2,3]. Type I IFN activity in SLE is associated with disease severity [1]. TNF was the first cytokine convincingly demonstrated to contribute to chronic inflammation in several autoimmune diseases, including rheumatoid arthritis (RA) and Crohn disease [4]. Accordingly, blockade of TNF activity has proven to be highly beneficial in the treatment of these diseases [5,6].

Blockade of TNF reduces the acute-phase reaction and decreases the local and systemic levels of inflammatory mediators in patients with RA (reviewed in [7]). However, the improvement varies between patients, and approximately 30% of RA patients fail to respond to this therapy. It has been suggested that TNF suppresses IFNα production by inhibiting both the generation of plasmacytoid dendritic cells (pDCs) and their IFNα secretion [8,9]. Accordingly, it was shown that TNF blockade in systemic-onset juvenile idiopathic arthritis (SoJIA) patients, which resulted in a poor or fair clinical response [10]. is associated with a higher expression of IFN response genes [9]. The *in vivo *IFN bioactivity was determined by the measurement of the expression of type I IFN response genes in the peripheral blood cells. Similar findings were made for patients with primary Sjögren syndrome (SS) who were treated with a TNF antagonist [11] in which no evidence of efficacy of infliximab was observed [12]. Here, the type I IFN bioactivity in the blood was measured in an indirect manner, based on the use of a bioassay in which a serum sample is tested to induce the expression of IFN response activity.

Since the finding of an increased IFN response gene activity after TNF blockade was based on studies in diseases in which the clinical response to therapy was shown not to be optimal, we were interested to know whether this effect also applied to diseases that showed a good clinical response. Therefore, we aimed to determine the effect of TNF blockade on the type I IFN response gene activity in RA patients, for approximately two thirds of whom TNF-blocking therapy is effective. Previously, we and others demonstrated increased type I IFN response gene activity in the peripheral blood cells of approximately 50% of anti-TNF treatment-naive RA patients [13]. This analysis was based on the measurement of the expression of a set of 34 type I IFN response genes. Accordingly, others demonstrated increased levels of IFNα in serum of a subset of RA patients [14]. Here, we first studied whether TNF blockade in RA led to a consistent increase in type I IFN response gene activity as was reported for SoJIA and SS. Subsequently, we determined whether anti-TNF-induced changes in IFN response activity were associated with the clinical outcome of TNF blockade in RA.

## Materials and methods

### Patients

Consecutive patients with RA according to the American College of Rheumatology criteria were enrolled in the study at the outpatient clinic of the Academic Medical Center (AMC) in Amsterdam over a period of 1 year. Inclusion criteria were 18 to 85 years of age, a failure of at least two disease-modifying anti-rheumatic drugs (including methotrexate [MTX]), and active disease (disease activity score using 28 joint counts [DAS28] of at least 3.2). Patients with a history of an acute inflammatory joint disease of different origin or previous use of a TNF-blocking agent were excluded. Patients were on stable, maximally tolerable MTX treatment. Whole blood samples (2.5 mL) were obtained using PAXgene tubes (PreAnalytiX GmbH, Hilden, Germany) from 33 RA patients prior to initiation of anti-TNF therapy with infliximab (3 mg/kg intravenously at baseline and weeks 2 and 6 and subsequently every 8 weeks). After 4, 8, 12, and 16 weeks of treatment, another PAXgene tube was obtained. All patients gave written informed consent, and the study protocol was approved by the Medical Ethics Committee (AMC). After 16 weeks of treatment, clinical response was assessed using the European League Against Rheumatism (EULAR) response criteria [15,16] as well as the reduction in DAS28 (response defined by a decrease in DAS28 of at least 1.2) [17]. According to the EULAR response criteria, 6 of the 33 patients had a poor response whereas 12 patients displayed a good response to treatment. An overview of the patients' characteristics is presented in Table 1.

**Table 1:** Characteristics of patients at baseline

	Array analysis (n = 15)	qPCR analysis (n = 18)
Age, years	51 (39-55)	58 (51-69)
Gender, female/male	7/8	14/4
Disease characteristics		
DAS28	5.6 (4.6-7.0)	5.7 (5.0-6.6)
C-reactive protein, mg/dL	8 (6-22)	13 (5-44)
Erythrocyte sedimentation rate	25 (12-41)	32 (16-47)
ACPA titer, U/mL	100 (15-595)	541 (121-1,805)
IgM RF titer, U/mL	28 (14-133)	67 (22-182)
Disease duration, months	77 (29-240)	65 (36-1,992)
Erosions	n = 13	n = 15
Medication		
Methotrexate dose, mg/week	25 (20-30)	21 (15-25)
Prednisone	n = 2	n = 5
NSAID	n = 7	n = 12

Values are presented as median (interquartile range 25 to 75) unless indicated otherwise. ACPA, anti-citrullinated protein antibodies; DAS28, disease activity score using 28 joint counts; NSAID, nonsteroidal anti-inflammatory drug; qPCR, quantitative polymerase chain reaction; RF, rheumatoid factor.

### Blood sampling for RNA isolation

Blood (2.5 mL) was drawn in PAXgene blood RNA isolation tubes (PreAnalytiX GmbH) and stored at -20°C. Tubes were thawed for 2 hours at room temperature prior to RNA isolation. Next, total RNA was isolated using the PAXgene RNA isolation kit according to the manufacturer's instructions, including a DNAse (Qiagen, Venlo, The Netherlands) step to remove genomic DNA. Quantity and purity of the RNA were tested using the NanoDrop spectrophotometer (NanoDrop Technologies, Inc., Wilmington, DE, USA).

### Microarray data

In 15 patients, the baseline expression of type I IFN-regulated genes was determined using cDNA microarrays and compared with levels 1 month after treatment. Therefore, we used 43 K cDNA microarrays (from the Stanford Functional Genomics Facility [18]) printed on aminosilane-coated slides containing approximately 20,000 unique genes. DNA spots were UV-crosslinked to the slide using 150 to 300 mJ. Sample preparation and microarray hybridization were performed as described previously [13,19]. Data storage and filtering were performed using the Stanford Microarray Database [20,21] as described previously [22]. Raw data (log2) can be downloaded from the publicly accessible Stanford database website [21]. In addition, data are stored in the Gene Expression Omnibus [23] [GEO:GSE19821].

### Interferon response gene set

Previously, we showed that a prominent cluster of highly correlated type I IFN response genes is upregulated in a subgroup of biological-naive RA patients compared with healthy controls [13]. A gene set consisting of 34 type I IFN response genes was obtained from these data. A smaller IFN gene set consisting of 15 genes was selected for validation analysis using a BioMark™ Real-Time PCR [Polymerase Chain Reaction] System (Fluidigm Corporation, South San Francisco, CA, USA). Detailed information of the gene lists is presented in Table 2.

**Table 2:** Interferon response gene/transcript sets used in this study

IFN set (34 genes)	Validation (15 genes)	Symbol	NCBI mRNA accession number	Name
X		AA075725	AA075725.1	None
X		AA142842	AA142842.1	None
X		AI347124	AI347124.1	None
X		ATF3	NM_001030287	Activating transcription factor 3
X		EIF2AK2	NM_001135651	Eukaryotic translation initiation factor 2-alpha kinase 2
X	X	EPSTI1	NM_001002264	Epithelial stromal interaction 1 (breast)
X		Hs.128576	NM_001135993	CDNA FLJ90394 fis, clone NT2RP2005632
X		Hs.97872	AI821640	Transcribed locus
X		IFI16	NM_005531	Interferon, gamma-inducible protein 16
X	X	IFI35	NM_005533	Interferon-induced protein 35
X	X	IFI44L	NM_006820	Interferon-induced protein 44-like
X	X	IFI6	NM_002038	Interferon, alpha-inducible protein 6
X	X	IFIT1	NM_001548	Interferon-induced protein with tetratricopeptide repeats 1
X		IFIT2	NM_001547	Interferon-induced protein with tetratricopeptide repeats 2
X	X	IFITM1	NM_003641	Interferon-induced transmembrane protein 1 (9-27)
X		IL1RN	NM_000577	Interleukin 1 receptor antagonist
X	X	IRF2	NM_002199	Interferon regulatory factor 2
X		IRF7	NM_001572	Interferon regulatory factor 7
X	X	ISG15	NM_005101	ISG15 ubiquitin-like modifier
X	X	LGALS3BP	NM_005567	Lectin, galactoside-binding, soluble, 3 binding protein
X		MX1	NM_001144925	Myxovirus (influenza virus) resistance 1
X	X	MX2	NM_002463	Myxovirus (influenza virus) resistance 2 (mouse)
X	X	OAS1	NM_001032409	2',5' -oligoadenylate synthetase 1, 40/46 kDa
X	X	OAS2	NM_001032731	2' -5' -oligoadenylate synthetase 2, 69/71 kDa
X		PARP14	NM_017554	Poly (ADP-ribose) polymerase family, member 14
X		PLSCR1	NM_021105	Phospholipid scramblase 1
X		RNF213	NM_020914	Ring finger protein 213
X	X	RSAD2	NM_080657	Radical S-adenosyl methionine domain containing 2 (alias cig5)
X		RTP4	NM_022147	Receptor (chemosensory) transporter protein 4
X	X	SAMD9L	NM_152703	Sterile alpha motif domain containing 9-like
X	X	SERPING1	NM_000062	Serpin peptidase inhibitor, clade G (C1 inhibitor), member 1
X		TAP1	NM_000593	Transporter 1, ATP-binding cassette, sub-family B (MDR/TAP)
X		TNFAIP6	NM_007115	Tumor necrosis factor, alpha-induced protein 6
X		UBE2L6	NM_004223	Ubiquitin-conjugating enzyme E2L 6

IFN, interferon; NCBI, National Center for Biotechnology Information.

### Real-time quantitative polymerase chain reaction

RNA (0.5 μg) was reverse-transcribed into cDNA using the Revertaid H-minus cDNA synthesis kit (MBI Fermentas, St. Leon-Rot, Germany) according to the manufacturer's instructions. Real-time quantitative PCR (qPCR) was performed using an ABI Prism 7900HT Sequence detection system (Applied Biosystems, Foster City, CA, USA) using SybrGreen (Applied Biosystems). Primers were designed using Primer Express software and guidelines (Applied Biosystems), and used primer sequences are listed in Additional file 1. To calculate arbitrary values of mRNA levels and to correct for differences in primer efficiencies, a standard curve was constructed. Expression levels of target genes were expressed relative to *18SRNA*.

### BioMark™ Real-Time PCR System

The BioMark™ 48.48 Dynamic Array (Fluidigm Corporation) for real-time qPCR was used to simultaneously measure the expression of 15 IFN response genes (Table 2) in 47 samples (plus one negative control) in triplicate. The 47 samples were derived from 10 patients (five poor and five good EULAR responders) at baseline and 1, 2, 3, or 4 months after treatment. From two poor and one good responder patients, the 3-month time points are missing. This experiment was performed at the outsourcing company ServiceXS (Leiden, The Netherlands). Used pre-designed Taqman Gene Expression Assays are listed in Additional file 1. Expression levels of target genes were expressed relative to *18SRNA*.

### Statistical analysis

Data were analyzed using software programs GraphPad Prism 4 (GraphPad Software, Inc., San Diego, CA, USA) and SPSS version 14.0 (SPSS Inc., Chicago, IL, USA). Data were checked for normal (Gaussian) distribution. Paired *t *test analysis was used to compare pre- and post-treatment expression levels. Two-group comparisons were analyzed using unpaired *t *test or two-way analysis of variance (ANOVA), where appropriate. Data were considered significant with *P *values of less than 0.05.

## Results

### Differential effect of tumor necrosis factor blockade on type I interferon signature

**Figure 1:**
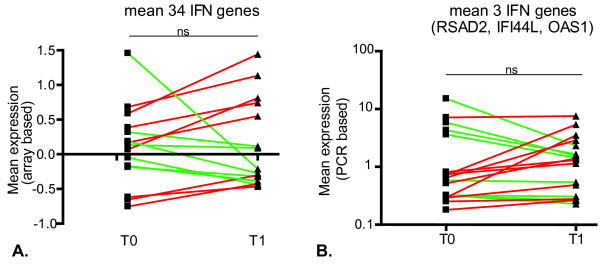
**Differential regulation of interferon (IFN) response genes upon tumor necrosis factor (TNF) blockade**. The expression levels of 34 type I IFN response genes were determined by cDNA microarray analysis in peripheral blood cells of 15 patients before (T0) and 1 month after (T1) anti-TNF treatment. **(a) **Subsequently, for each patient, the expression levels were averaged (note: data are in log2 space) and baseline levels were compared with post-treatment levels. The patients whose IFN response gene levels are induced after TNF blockade are indicated by red lines, and patients with a downregulation are indicated by green lines. Subsequently, the expression levels of three IFN response genes (*RSAD2*, *IFI44L*, and *OAS1*) were measured by quantitative real-time polymerase chain reaction (PCR) in an independent group of 18 patients. **(b) **The expression levels of the three genes were averaged, and baseline levels were compared with post-treatment levels. ns, not significant using a paired *t *test analysis.

Previously, we compared the gene expression profiles of peripheral blood cells of RA patients with those of healthy controls and found that a subgroup of RA patients has an increased expression of type I IFN response genes [13]. This increased expression in IFN response genes was highly variable between the individual RA patients and unrelated to medication and disease activity. In the present study, we studied the effect of TNF blockade on the transcription of type I IFN response genes. Therefore, we used the expression values of 34 type I IFN response genes (Table 2) (described previously [13]), which were averaged. Subsequently, baseline values were compared with post-treatment levels (Additional file 2). At the patient group level, there was no significant change in type I IFN response gene activity (Figure 1a). However, the regulation of IFN response genes upon TNF blockade was highly variable between patients. The variation was not related to gender or MTX dose or prednisone or nonsteroidal anti-inflammatory drug (NSAID) use.

To confirm these results in an independent cohort of 18 RA patients, three genes (*RSAD2*, *IFI44L*, and *OAS1*) that showed the best correlation (R > 0.9) with the mean expression value of the set of 34 type I IFN response genes were selected. The mean expression of the three genes was measured by real-time qPCR before and 1 month after infliximab therapy. Ten patients showed an increased expression of these three IFN response genes after TNF blockade, whereas in eight patients similar or decreased levels were observed (Figure 1b). The IFN regulation was independent of gender or MTX dose or prednisolone or NSAID use. Collectively, these results confirm findings from the microarray study and evidently demonstrate that the regulation of IFN response gene activity upon TNF blockade in RA is not as consistent as previously described for SoJIA [9] and SS [11].

### Change in type I interferon response gene activity is unrelated to baseline levels

Since the type I IFN response gene expression levels are already highly heterogeneous in biological-naive RA patients, we investigated whether the observed changes were related to the magnitude of IFN response gene expression prior to treatment. Therefore, the relationship between the extent of the baseline IFN response gene expression levels and its change after TNF blockade was tested. In the 15 patients, the baseline mean expression of the type I IFN gene set did not correlate with their corresponding change after treatment (Pearson R = -0.42, *P *= 0.12). This was confirmed in the validation group of 18 patients by using the mean expression levels of the three IFN response genes (*RSAD2*, *IFI44L*, and *OAS1*) measured by qPCR, although a trend toward significance was observed (Spearman R = -0.44, *P *= 0.064). These findings reveal that the type I IFN response gene expression profile prior to treatment is not associated with the direction of its change upon TNF blockade.

### Anti-tumor necrosis factor induced-interferon regulation and clinical response to treatment

**Figure 2:**
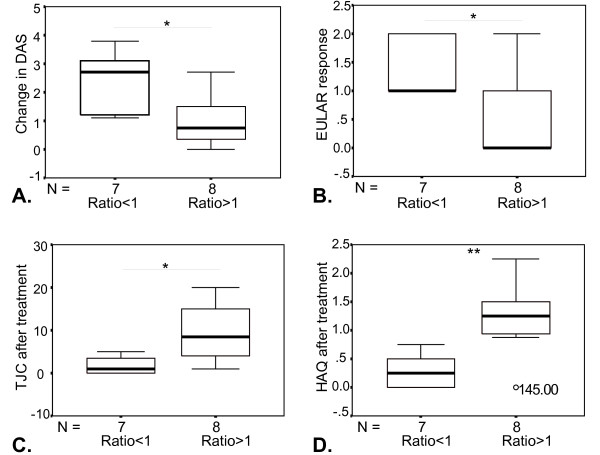
**Differential regulation of interferon (IFN) response genes upon tumor necrosis factor (TNF) blockade and clinical response to treatment**. Patients were divided into two groups (ratio < 1 and ratio > 1) on the basis of their IFN response upon TNF blockade and compared with each other with respect to clinical response to treatment. The ratio is determined by the T = 1/T = 0 expression levels of the IFN response genes as demonstrated in Figure 1a. Data are shown as box plots; each box represents the 25th to 75th percentiles. The lines inside represent the median, and the ends of the whiskers represent the smallest and largest observations. Patients with an upregulation in IFN response genes displayed a significantly (unpaired *t *test, * *P *< 0.05; **P < 0.01) worse clinical response to treatment as assessed by change in disease activity score (DAS) (DAS before treatment minus DAS 16 weeks after treatment) **(a)**, European League Against Rheumatism (EULAR) response **(b)**, tender joint count (TJC) **(c)**, and Health Assessment Questionnaire-Disability Index (HAQ) **(d) **after treatment.

Finally, we investigated whether the treatment-induced changes in type I IFN response gene expression levels were associated with clinical response to treatment. Therefore, the patients (n = 15) were divided into two groups on the basis of their change in mean expression level for the 34 IFN response genes (ratio > 1 and ratio < 1) as demonstrated in Figure 1a. Next, clinical parameters were compared between these two groups. Clinical response to treatment was determined after 16 weeks of treatment. Interestingly, the patients who showed an increase in type I IFN response gene expression levels after 1 month of treatment had a poor clinical response to treatment. This was reflected by less improvement in disease activity scores (*P *= 0.013) and higher tender joint counts (*P *= 0.015) and higher Health Assessment Questionnaire-Disability Index scores (*P *= 0.008) after treatment (Figure 2). Accordingly, all patients without an anti-TNF-induced increase in type I IFN gene activity had a good or moderate response to treatment as assessed by the EULAR response criteria (*P *= 0.018) (Figure 2). From a total of 29 patients, both the EULAR and the qPCR expression data were available for the three IFN response genes *RSAD2*, *IFI44L*, and *OAS1*. Analysis of the pre- versus post-treatment ration of *OAS1 *revealed that the change in gene expression of *OAS1 *is significantly associated with clinical response to treatment (*P *< 0.013).

**Figure 3:**
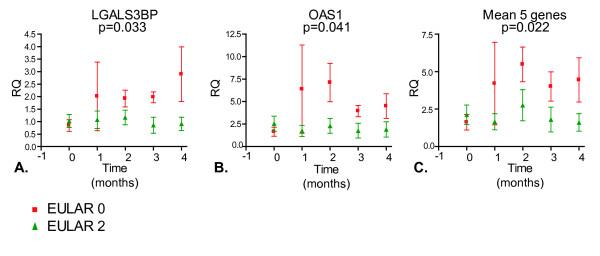
**Poor response to tumor necrosis factor blockade is accompanied by upregulation of interferon (IFN) response genes**. For five European League Against Rheumatism (EULAR) (0) poor responder (red) and five EULAR (2) good responder (green) patients, the expression levels of 15 IFN response genes were measured by quantitative real-time polymerase chain reaction (PCR) (BioMark™) at baseline and 1, 2, 3, and 4 months after treatment. From two poor and one good responder patients, the 3-month time points are missing. The IFN response gene expression levels during treatment were compared between the two clinical response groups by means of two-way analysis of variance test. Treatment-induced changes in the expression levels of two genes, *LGALS3BP ***(a) **and *OAS1 ***(b)**, were significantly different between the two response groups. **(c) **The mean expression level of five IFN response genes (*LGALS3BP*, *OAS1*, *Mx2*, *SERPING1*, and *OAS2*) showed the best significant difference between the two clinical response groups. Graphs show the mean and standard error of the mean expression levels for each clinical response group. RQ, relative quantity.

To determine whether the IFN response to TNF blockade was sustained over time, five EULAR good and five EULAR poor responders were selected and the expression levels of 15 IFN response genes (selected from the set of 34 genes used above, Table 2) were measured at baseline and 1, 2, 3, and 4 months after treatment by qPCR (Additional file 3). The expression levels were averaged for the individual patients, and the treatment-induced changes (ratio post- versus pre-treatment) in IFN response gene expression levels over time were compared between the two clinical response groups using two-way ANOVA. Overall, the IFN response genes showed an upregulation in the poor responder group, which was most prominent at 2 months after the start of therapy (data not shown). At the single-gene level, the increased expression in poor versus good responders reached significance for the *OAS1 *and *LGALS3BP *genes (Figure 3a, b). For three other IFN response genes (*Mx2*, *OAS2*, and *SERPING1*), a trend (*P *= approximately 0.06, data not shown) was observed toward increased expression in the poor responder patients. Combining these five genes (*OAS1*, *LGALS3BP*, *Mx2*, *OAS2*, and *SERPING1*) into one IFN response gene set improved the significance (Figure 3c). These data demonstrate that poor response to infliximab treatment is associated with treatment-induced increase in type I IFN response gene activity.

## Discussion

In this study, we demonstrated that blockade of the inflammatory cytokine TNF in RA patients modulates the expression of IFN response gene activity in a heterogeneous manner. The data revealed that some RA patients display a treatment-induced increased expression of type I IFN response genes whereas others display no effect or a small decrease. We provided evidence that the treatment-induced change in expression levels of IFN response genes is associated with the EULAR response rate at 16 weeks after the start of infliximab treatment. RA patients who revealed an increased IFN response gene expression profile after 1 to 2 months of anti-TNF treatment exhibited a poor clinical response. No association between clinical response to infliximab treatment and baseline IFN response gene activity was found.

IFNs are known for their immune regulatory properties. Previously, we provided evidence for an increased expression of type I IFN response genes in a subset of patients with RA [13]. Upregulation of type I IFN response genes has been reported in peripheral blood cells of (a subset of) patients with other autoimmune diseases, like SLE [1,24-26], dermatomyositis [27], and multiple sclerosis [22]. Type I IFNs (IFNαβ) exert broad dual effects on the immune system, reflecting both immune-stimulatory and immune-suppressive activities. Immune-stimulatory activities relate to the activation of myeloid dendritic cells, chemokines, chemokine receptors, costimulatory molecules (CD40, CD80, and CD86), and humoral responses. Immune-suppressive effects are reflected by Th2 cell skewing and anti-proliferative and pro-apoptotic effects. According to their dual effect on immunity, their role in disease may range from detrimental to beneficial. Although the anti-TNF-induced increase in IFN response activity might be an epiphenomenon related to the effect of TNF blockade, it is tempting to speculate on a role of increased IFN bioactivity in the deteriorating clinical effects. The association between an increase in type I IFN response gene activity and poor response to anti-TNF treatment may suggest a harmful role for type I IFN bioactivity in RA or, alternatively, a failed attempt to counter-regulate inflammation.

Clinical experience revealed that a fraction of patients treated with TNF antagonists developed (increased) anti-dsDNA antibodies, in some cases with a concomitant lupus-like syndrome [28,29]. From a total of 18 of the 33 patients we studied, levels of anti-citrullinated protein antibody, rheumatoid factor, and double-stranded DNA autoantibody, determined before and 24 weeks after TNF blockade, were available. The expression level of IFN response genes was not related to antibody levels at baseline nor was the activation of the IFN response genes related to the drug-induced formation of antibodies (data not shown [30]).

The differences between the effects of TNF blockade on IFN response activity between the studies in SS [11] and SoJIA [9], on the one hand, and our studies in RA and spondyloarthritis (SpA) [31], on the other, could have their origin in differences in design between the studies, such as the use of infliximab in RA versus etanercept in SS, and the different readout systems used. For example, the SS study was based on an indirect reporter cell assay to measure type I IFN activity in plasma, whereas in the SoJIA and RA studies, measurement of IFN response gene activity in the peripheral blood cells was measured. Using the reporter assay, we previously demonstrated that the serum type I IFN bioactivity was increased in SpA patients treated with etanercept whereas it was transiently declined by infliximab [31]. This is suggestive for differential effects of etanercept and infliximab on IFN response activity, although the direct consequence for the IFN response signature in the peripheral blood cells has not been tested. In an attempt to find an explanation for the apparent discrepancies in IFN response gene activity related to clinical response to infliximab, we learned that in both RA and SS [11,12] an increased IFN response activity is associated with a poor clinical response.

The inter-individual differences in anti-TNF-induced IFN response may be the result of differential regulatory processes. Evidence that TNF blockade may exert both inhibitory and activating effects on IFN response activity is available. *In vitro *experiments suggested that endogenous secretion of TNF by pDCs represents a negative feedback on IFN production [9]. Whereas this finding suggested that TNF displays counteracting effects on IFN response activity, others have reported that TNF initiates an IRF1-dependent autocrine loop leading to sustained expression of STAT1-dependent type I IFN response genes [32]. Hence, the divergent outcome of the IFN response activity might be a consequence of differences in the relative contribution of each of these processes in the regulation of IFN response activity. Alternatively, genetic variation in the type I IFN biology could underlie the variation in response activity. Single-nucleotide polymorphisms in several transcription factors involved the type I IFN pathway (for example, *IRF5*, *Tyk2*, and *STAT4*) have recently been associated with a number of autoimmune diseases, including SLE [33,34] and RA [35-37]. Future studies are needed to unravel the mechanism behind the divergent alterations in IFN response gene activity upon TNF blockade. Since the current information on the differential IFN response gene activity does not have predictive value to identify responders before the start of therapy, detailed insight in the regulatory processes that underlie this effect might be helpful to identify such biomarkers. Therefore, *in vitro *studies with blood cells that are treated with a TNF blocker might be useful.

## Conclusions

In summary, this study shows that there is a large variation between RA patients in the change of IFN response gene expression levels during TNF blockade. The change in IFN response genes is unrelated to baseline expression levels. Interestingly, treatment-induced increase of IFN response gene activity is associated with poor clinical response to infliximab treatment. Additional studies in larger patient cohorts should reproduce and confirm these findings.

## Abbreviations

AMC: Academic Medical Center; ANOVA: analysis of variance; DAS28: disease activity score using 28 joint counts; EULAR: European League Against Rheumatism; IFN: interferon; MTX: methotrexate; NSAID: nonsteroidal anti-inflammatory drug; PCR: polymerase chain reaction; pDC: plasmacytoid dendritic cell; qPCR: quantitative polymerase chain reaction; RA: rheumatoid arthritis; SLE: systemic lupus erythematosus; SoJIA: systemic-onset juvenile idiopathic arthritis; SpA: spondyloarthritis; SS: Sjögren syndrome; TNF: tumor necrosis factor.

## Competing interests

PT has served as a consultant for Abbott (Abbott Park, IL, USA), Amgen (Thousand Oaks, CA, USA), Centocor (Horsham, PA, USA), Schering-Plough Corporation (Kenilworth, NJ, USA), UCB (Brussels, Belgium), and Wyeth (Madison, NJ, USA). The VU University Medical Center has filed a patent application (patent file number P086657EP00, 'Predicting clinical response to treatment with a soluble TNF-antagonist or TNF, or a TNF receptor agonist'). The other authors declare that they have no competing interests.

## Authors' contributions

LvB helped to conceive, design, and perform the experiments, participated in analysis and interpretation of data, and helped to write the paper. CW helped to conceive, design, and perform the experiments, participated in patient inclusion and disease activity measure, and helped to write the paper. PT helped to conceive and design the experiments and participated in patient inclusion and disease activity measure. CV helped to conceive and design the experiments, participated in analysis and interpretation of data, and helped to write the paper. TvdPK participated in analysis and interpretation of data and analysis tools. TC, DB, and BD participated in patient inclusion, disease activity measurements and clinical laboratory analyses. FR helped to perform the experiments. All authors read and approved the final manuscript.

## Supplementary Material

Additional file 1 **Information on real-time PCR assays**. Primer sequences for quantitative real-time PCR and Pre-designed Taqman Gene Expression Assays used for Fluidigm's BioMark™ Real-Time PCR System. Click here for file

Additional file 2 **Microarray data values of the individual 34 IFN response genes**. Microarray data values (in log2) of the 34 IFN response genes measured in 15 RA patients before and one month after infliximab treatment including EULAR response. Click here for file

Additional file 3 **qPCR values of the individual 15 IFN response genes**. Gene expression values of 15 IFN response genes measured by Fluidigm's BioMark™ Real-Time PCR System. Expression values were measured before start of treatment, one, two, three and four months after start of therapy in 5 EULAR good responder patients and 5 EULAR poor responder patients. Click here for file
